# Determining the Upper-Bound on the Code Distance of Quantum Stabilizer Codes Through the Monte Carlo Method Based on Fully Decoupled Belief Propagation

**DOI:** 10.3390/e27090940

**Published:** 2025-09-09

**Authors:** Zhipeng Liang, Zicheng Wang, Zhengzhong Yi, Fusheng Yang, Xuan Wang

**Affiliations:** 1 School of Computer Science and Technology, Harbin Institute of Technology (Shenzhen), Shenzhen 518055, China; liangzhipenghitsz@163.com (Z.L.); wzc4370@163.com (Z.W.); hityfs@163.com (F.Y.); 2 Hefei National Laboratory, University of Science and Technology of China, Hefei 230088, China; 3 Hefei National Research Center for Physical Sciences at the Microscale and School of Physical Sciences, University of Science and Technology of China, Hefei 230026, China; 4 Shanghai Research Center for Quantum Science and CAS Center for Excellence in Quantum Information and Quantum Physics, University of Science and Technology of China, Shanghai 201315, China

**Keywords:** quantum stabilizer code, code distance, quantum XYZ product code, Z-type Tanner-graph-recursive-expansion code, Chamon code

## Abstract

The code distance is a critical parameter of quantum stabilizer codes (QSCs), and determining it—whether exactly or approximately—is known to be an NP-complete problem. However, its upper bound can be determined efficiently by some methods such as the Monte Carlo method. Leveraging the Monte Carlo method, we propose an algorithm to compute the upper bound on the code distance of a given QSC using fully decoupled belief propagation combined with ordered statistics decoding (FDBP-OSD). Our algorithm demonstrates high precision: for various QSCs with known distances, the computed upper bounds match the actual values. Additionally, we explore upper bounds for the minimum weight of logical *X* operators in the Z-type Tanner-graph-recursive-expansion (Z-TGRE) code and the Chamon code—an XYZ product code constructed from three repetition codes. The results on Z-TGRE codes align with theoretical analysis, while the results on Chamon codes suggest that XYZ product codes may achieve a code distance of $O(N^{2/3})$, which supports the conjecture of Leverrier et al.

## 1. Introduction

Quantum computing can solve certain problems that are intractable for classical computers under limited resources and time [1]. However, qubits—the basic units of quantum computing—are susceptible to environmental noise. This susceptibility compromises computation results and impedes the realization of quantum computing’s potential. Fortunately, Shor [2] and Steane [3] proposed quantum error-correcting codes (QECCs) in 1995, enabling reliable quantum computing.

QSCs [4] are an important class of quantum error-correcting codes. When designing a new QSC, it is necessary to precisely determine its code distance, since the code distance determines the number of physical qubits that can be reliably corrected. Generally, there are three methods to precisely determine the code distance of a QSC: the theoretical proof method, the linear programming method, and brute-force search. For some QSCs, such as the planar surface code [5,6], the XZZX surface code [7], and *D*-dimensional (D≥2) toric codes [8,9,10,11], their code distance can be determined by the properties of their topological structure. For QSCs with a systematic construction method, such as the hypergraph product code [12], we can also theoretically prove their code distance by using the construction method. For some QSCs whose code distance cannot be theoretically determined by the above two methods, it can be computed using linear programming [13] or brute-force search. However, both approaches are computationally intensive, exhibiting exponential time complexity. It has been theoretically proven that computing the code distance of a QSC, either exactly or approximately, is NP-complete [14]. However, the upper bound on the code distance can still be efficiently determined using methods such as Monte Carlo.

The Monte Carlo method [15] is widely used to efficiently approximate solutions to complex problems through stochastic simulations. In quantum error correction, it enables rapid determination of upper bounds on the code distance for certain QSCs [16,17]. The core procedure is as follows: First, perform multiple error-correction simulations on the target code. Second, record the weights of recovered errors that correspond to nontrivial logical operators. Third, take the minimum weight as the upper bound on the code distance. This method imposes no strict constraints on the type of decoder, provided the chosen decoder is applicable and effective for the QSC under test. However, when designing novel QSCs—where decoder compatibility is often unknown—a highly general, performant, and efficient decoder becomes essential for practical Monte Carlo-based distance estimation.

Belief propagation (BP) is a low-complexity decoder applicable to all QSCs. However, its error-correction performance is typically poor due to short cycles [18]. As shown in Ref. [16], ordered statistics decoding (OSD) [19] can enhance BP performance when BP fails to converge. Studies further demonstrate that BP-OSD, the combination of conventional BP with OSD, achieves strong error-correction performance on many Calderbank–Shor–Steane (CSS) quantum low-density parity-check (QLDPC) codes [20,21]. Thus, BP-OSD is highly general, high-performance, and efficient. Nevertheless, for non-CSS codes, conventional binary BP (even with OSD) delivers unsatisfactory performance, particularly under Y-biased noise.

The decoding algorithm employed here is the fully decoupled BP combined with OSD (FDBP-OSD), which was proposed in our previous work [22]. There are two major advantages of this decoding algorithm: (1) it achieves satisfactory accuracy for both CSS and non-CSS codes, and (2) compared with conventional binary BP [23,24], FDBP exhibits higher convergence rates and decoding accuracy.

In this paper, we leverage the Monte Carlo method to propose an algorithm for determining upper bounds on the code distance of QSCs using FDBP-OSD. Our analysis proceeds in three stages: First, we validate the algorithm by computing upper bounds for QSCs with known code distances (e.g., the planar surface code, the XZZX surface code, and the toric code), confirming its effectiveness. The results demonstrate high precision—the computed upper bounds match the actual distances. Second, for Z-TGRE codes [25], the algorithm-determined minimum weight of logical *X* operators aligns with theoretical analysis. Third, we explore Chamon codes—the XYZ product [26] of three repetition codes with block lengths $n_{1}$, $n_{2}$, and $n_{3}$, whose code distance remains poorly understood. Our results show that when $n_{1}=n_{2}=n_{3}=L$, its upper bound of the code distance is 2L, and when $n_{1}=L-1$, $n_{2}=L$, $n_{3}=L+1$, its upper bound of the code distance is L(L−1). This results implies that the code distance of XYZ product codes can very likely achieve $O(N^{2/3})$ [26]. We emphasize that, since FDBP-OSD is a highly general, high-performance, and fast decoding algorithm for both CSS and non-CSS codes, when designing new QSCs and their code distance is hard to compute, the algorithm is a useful method to quickly determine the upper bound of their code distance.

The rest of the paper is organized as follows: In Section 2, we introduce some preliminaries, including the quantum stabilizer code, the Z-TGRE code, and the XYZ product code. Section 3 introduces methods to determine the upper bound of the code distance of QSCs through the Monte Carlo method based on FDBP-OSD. The simulation results are presented in Section 4. In Section 5, we conclude our work.

## 2. Preliminaries

### 2.1. Quantum Stabilizer Code

This section briefly introduces the basic concept of QSCs. QSCs constitute an important class of QECCs and can be viewed as the quantum counterpart of classical linear error-correcting codes.

Given an [n,k,d] QSC *C*, its code space $Q_{C}$ is a $2^{k}$-dimensional subspace of the Hilbert space $H_{2}^{⊗n}$, which is stabilized by a set of Pauli operators, $S\in G_{n}$, where $G_{n}=G_{1}^{⊗n}$ and $G_{1}=\left\{\pm I,\;\pm iI,\;\pm X,\;\pm iX,\;\pm Y,\;\pm iY,\;\pm Z,\;\pm iZ\right\}$ is the single-qubit Pauli group. Formally,

(1) $$Q_{C}=\left\{|\varphi \rangle \in {\left(H^{2}\right)}^{⊗n}:S|\varphi \rangle =|\varphi \rangle ,\forall S\in S\right\}$$

The set S is referred to as the stabilizer group, which is Abelian and can be generated by n−k-independent *n*-qubit Pauli operators $S_{1},\cdots ,S_{n-k}\in G_{n}$, namely, $S=\langle S_{1},\cdots ,S_{n-k}\rangle$. The code space is the common eigenspace corresponding to the +1 eigenvalue of all stabilizer generators. Therefore, providing a set of stabilizer generators $S_{1},\cdots ,S_{n-k}$ of code *C* is equivalent to explicitly providing the code space $Q_{C}$.

Let $s=\left(s_{1},\cdots ,s_{n-k}\right)$ be a binary vector corresponding to an *n*-qubit Pauli error $E\in G_{n}$; if *E* anti-commutes with the stabilizer generator $S_{i}$, $s_{i}=1$; otherwise, $s_{i}=0$.

In quantum information theory, the single-qubit Pauli operators *X*, *Y*, *Z* and the identity operator *I* can be represented by two bits as follows:

(2) I→(0,0),X→(1,0),Z→(0,1),Y→(1,1)

Thus, any error $E\in G_{n}$ can be represented by a binary vector $e=\left(e_{x}|e_{z}\right)$ of length 2n, which is referred to as the symplectic representation of *E*. Based on the symplectic representation, the binary parity-check matrix *H* of an [[n,k,d]] QSC is a block matrix with dimension $\left(n-k\right)\times 2n$, which consists of two $\left(n-k\right)\times n$ binary matrices $H_{x}$ and $H_{z}$, where

(3) $$H=(H_{x}|H_{z}).$$

And the syndrome *s* of *E* is computed as follows:

(4) $$s=(H_{x}\cdot e_{z}+H_{z}\cdot e_{x})\;mod\;2$$

For a QSC, if its stabilizer generators can be divided into two parts, each of which only contains either *X*-type or *Z*-type Pauli operators, it is a CSS code; otherwise, it is a non-CSS code. In this way, the parity check matrix of a CSS code can be written as

(5) $$H=\left(\begin{array}{cc} H_{x} & 0\\ 0 & H_{z} \end{array}\right)$$

where $H_{x}$ and $H_{z}$ both have *n* columns and the commutation condition satisfies $H_{x}H_{z}^{T}=0$.

The weight of an operator $P\in G_{n}$ is defined as the number of single-qubit Pauli operators that it contains, and it is denoted as $wt\left(P\right)$. For instance, $wt\left(I_{1}X_{2}Y_{3}Z_{4}\right)=3$.

The logical operators of a QSC are the set of operators in $G_{n}$ which commute with all elements in S but are not in S. More precisely, the logical operators are the elements of C(S)∖S, where C(S) is the centralizer of S and is defined as $C\left(S\right)=\left\{P\in G_{n}:SP=PS,\;\forall S\in S\right\}$. For an [[n,k,d]] QSC, we can find *k* pairs of logical operators ${\left({\bar{X}}_{j},{\bar{Z}}_{j}\right)}_{j=1,\cdots ,k}$ such that ${\bar{X}}_{i}{\bar{Z}}_{j}={\left(-1\right)}^{\delta_{ij}}{\bar{Z}}_{j}{\bar{X}}_{i}$, where δ is the Kronecker delta, which means that for the same pair of logical operators ${\bar{X}}_{j},{\bar{Z}}_{j}$, they are anti-commute, but they commute with other pairs of logical operators. We can see that $C\left(S\right)=\left\{S_{1},\cdots ,S_{n-k},X_{1},Z_{1},\cdots ,X_{k},Z_{k}\right\}$. The code distance *d* is defined as the minimum weight of the logical operators, where

(6) $$d=\min_{L\in C(S)\setminus S}wt(L)$$

which determines the number of qubits, *t*, which the code *C* can reliably correct, namely $t=\left\lfloor \frac{d-1}{2}\right\rfloor$. In general, there are three methods to precisely compute the code distance of a QSC—the theoretical proof method, the linear programming method [13], and brute-force search. However, only a part of QSCs’ code distance can be theoretically proved. For some QSCs whose code distance cannot be theoretically determined, it can be computed using linear programming [13] or brute-force search. However, both approaches are computationally intensive, exhibiting exponential time complexity. Although theoretically proven to be NP-complete [14] (whether computed exactly or approximately), the code distance of QSCs can still be efficiently bounded from more efficient methods such as the Monte Carlo method.

### 2.2. Z-Type Tanner-Graph-Recursive-Expansion Code

**Figure 1:**
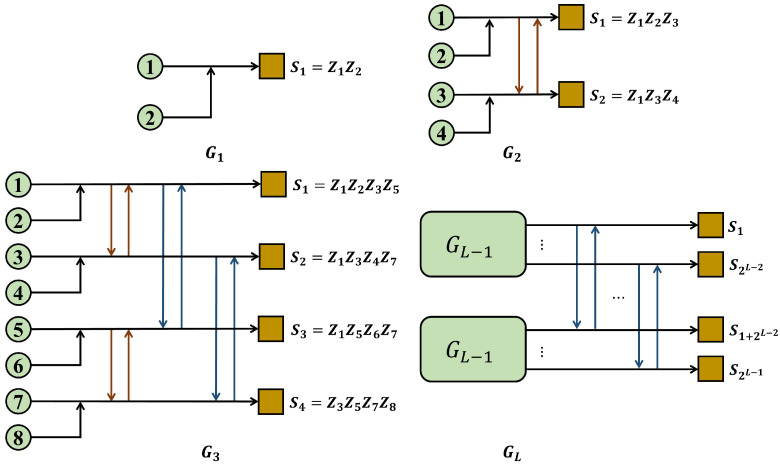
The Tanner graph recursive expansion of Z-TGRE codes. The arrow means that the corresponding variable node (the qubit) starts from 1 in the corresponding check node (the stabilizer) and ends with 8. The variable is numbered from 1 to $N=2^{L}$. $G_{1}$ is the primal Tanner graph used for recursive expansion. $G_{2}$ is the expanded Tanner graph according to the recursive expansion of two primal Tanner graphs of $G_{1}$. $G_{3}$ is the expanded Tanner graph according to the recursive expansion of two $G_{2}$ graphs. $G_{L}$ is the expanded Tanner graph according to recursive expansion of two $G_{L-1}$ graphs.

In our previous work [25], we propose a new class of quantum stabilizer codes named the Z-TGRE code, which is obtained by recursively expanding a Tanner graph, and it has a constant coding rate of 0.5 but can only correct Pauli-*X* and -*Y* errors. The way to expand the Tanner graph of the Z-TGRE code is shown in Figure 1.

The code length of the Z-TGRE code is $N=2^{L}$, and our theoretical analysis shows that, if *L* is an even number, the minimum weight of its logical *X* operators is $d_{x}=\log N$; if *L* is an odd number, $d_{x}=\log N+1$. Readers can see [25] for more detail.

### 2.3. XYZ Product Code

The XYZ product [26] is a three-fold variant of the hypergraph product code construction, which yields non-CSS QSCs. To better understand the XYZ product code, we should first introduce how to describe a CSS code in terms of chain complexes and the hypergraph product code.

A chain complex C of length *L* is a collection of L+1 vector spaces $C_{0}\cdots C_{L}$ and *L* linear maps $\partial_{i}:\;C_{i}⟶C_{i+1}$ (0≤i≤L−1), where

(7) $$C=C_{0}\overset{\partial_{0}}{⟶}C_{1}\overset{\partial_{1}}{⟶}\cdots \overset{\partial_{i-1}}{⟶}C_{i}\overset{\partial_{i}}{⟶}C_{i+1}\overset{\partial_{i+1}}{⟶}\cdots \overset{\partial_{L-1}}{⟶}C_{L}$$

which satisfies $\partial_{i+1}\partial_{i}=0$.

A chain complex C with length 2 naturally defines a CSS code C(C), namely

(8) $$C=F_{2}^{m_{z}}\overset{H_{z}^{T}}{⟶}F_{2}^{n}\overset{H_{x}}{⟶}F_{2}^{m_{x}}$$

where the commutation condition $H_{x}H_{z}^{T}=0$ is naturally satisfied.

It is easy to see that a classical code C=kerH similarly corresponds to a chain complex with length 1, namely

(9) $$F_{2}^{n}\overset{H}{⟶}F_{2}^{m}$$

The hypergraph product is using two classical codes $C_{1}=kerH_{1}$ and $C_{2}=kerH_{2}$ to construct a CSS code C (where $H_{i}$, i∈{1,2}, represents the parity check matrices of size $m_{i}\times n_{i}$ of codes $C_{i}$), which corresponds to the following length-2 chain complex:

(10) $$F_{2}^{m_{1}\times n_{2}}\overset{H_{z}^{T}}{\to }F_{2}^{n_{1}\times n_{2}}⊕F_{2}^{m_{1}\times m_{2}}\overset{H_{x}}{\to }F_{2}^{n_{1}\times m_{2}}$$

where $H_{x}=1_{n_{1}}⊗H_{2}⊕H_{1}^{T}⊗1_{m_{2}}$ and $H_{z}={\left(H_{1}^{T}⊗1_{n_{2}}⊕1_{m_{1}}⊗H_{2}\right)}^{T}$.

The XYZ product code construction is a variant of the hypergraph product code construction, which uses three classical codes to construct a non-CSS code. Specifically, given three parity check matrices $H_{i}$ of size $m_{i}\times n_{i}$$\left(i=1,2,3\right)$, the stabilizer generator matrix S of the corresponding XYZ product code is

(11) $$S=\left[\begin{array}{c} X^{\left(H_{1}⊗1_{n_{2}}⊗1_{n_{3}}\right)},Y^{\left(1_{m_{1}}⊗H_{2}^{T}⊗1_{n_{3}}\right)},Z^{\left(1_{m_{1}}⊗1_{n_{2}}⊗H_{3}^{T}\right)},I^{\left(m_{1}n_{2}n_{3}\times n_{1}m_{2}m_{3}\right)}\\ Y^{\left(1_{n_{1}}⊗H_{2}⊗1_{n_{3}}\right)},X^{\left(H_{1}^{T}⊗1_{m_{2}}⊗1_{n_{3}}\right)},I^{\left(n_{1}m_{2}n_{3}\times m_{1}n_{2}m_{3}\right)},Z^{\left(1_{n_{1}}⊗1_{m_{2}}⊗H_{3}^{T}\right)}\\ Z^{\left(1_{n_{1}}⊗1_{n_{2}}⊗H_{3}\right)},I^{\left(n_{1}n_{2}m_{3}\times m_{1}m_{2}n_{3}\right)},X^{\left(H_{1}^{T}⊗1_{n_{2}}⊗1_{m_{3}}\right)},Y^{\left(1_{n_{1}}⊗H_{2}^{T}⊗1_{m_{3}}\right)}\\ I^{\left(m_{1}m_{2}m_{3}\times n_{1}n_{2}n_{3}\right)},Z^{\left(1_{m_{1}}⊗1_{m_{2}}⊗H_{3}\right)},Y^{\left(1_{m_{1}}⊗H_{2}⊗1_{m_{3}}\right)},X^{\left(H_{1}⊗1_{m_{2}}⊗1_{m_{3}}\right)} \end{array}\right]$$

**Figure 2:**
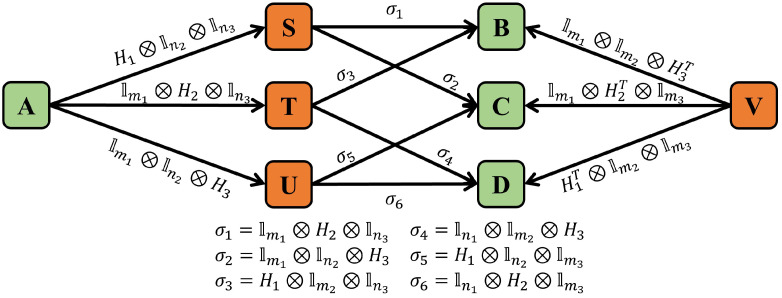
“Chain complex” representation of the XYZ product code.

Each row of S corresponds to a stabilizer generator. Here the notation $P=P^{H}\;\left(P\in \left\{X,Y,Z\right\}\right)$ denotes a Pauli tensor, which means that for any entry of matrix *H*, if it is 1, P has a Pauli operator *P* at the corresponding position and an identity operator *I* otherwise. Figure 2 shows the “chain complex” representation of the corresponding XYZ product code.

A, B, C, and D are vector spaces which index the qubits, and S, T, U and V are vector spaces which index the stabilizer generators, namely

(12) $$\begin{array}{cc} \; & A\in F_{2}^{n_{1}\times n_{2}\times n_{3}},\;B\in F_{2}^{m_{1}\times m_{2}\times n_{3}},\\ \; & C\in F_{2}^{m_{1}\times n_{2}\times m_{3}},\;D\in F_{2}^{n_{1}\times m_{2}\times m_{3}} \end{array}$$

and

(13) $$\begin{array}{cc} \; & S\in F_{2}^{m_{1}\times n_{2}\times n_{3}},\;T\in F_{2}^{n_{1}\times m_{2}\times n_{3}},\\ \; & U\in F_{2}^{n_{1}\times n_{2}\times m_{3}},\;V\in F_{2}^{m_{1}\times m_{2}\times m_{3}} \end{array}$$

It can be seen that the code length is $N=n_{1}n_{2}n_{3}+m_{1}m_{2}n_{3}+m_{1}n_{2}m_{3}+n_{1}m_{2}m_{3}$. As for the dimension of an XYZ product code, we need to find the number of independent stabilizer generators, and readers can see Ref. [26] for more detail.

In Ref. [26], Leverrier et al. found that this code family includes codes whose minimum distance is $O(N^{2/3})$. However, no one has proved it. The simplest instance of the XYZ product code is the Chamon code [27], which is the XYZ product of three repetition codes with block lengths $n_{1}$, $n_{2}$, and $n_{3}$, whose code distance has not been understood well. In Section 4, employing the Monte Carlo method based on FDBP-OSD, we show that when $n_{1}=n_{2}=n_{3}=L$, its upper bound of the code distance is 2L, and when $n_{1}=L-1$, $n_{2}=L$, and $n_{3}=L+1$, its upper bound of the code distance is L(L−1). The results implies that the code distance of the XYZ product code can very likely achieve $O(N^{2/3})$.

## 3. Determining the Upper Bound of Code Distance

It has been theoretically proven that computing the code distance of a QSC, either exactly or approximately, is NP-complete [14]. However, the upper bound on the code distance can still be efficiently determined using methods such as Monte Carlo.

This section first introduces the general idea of determining the upper bound of code distance using the Monte Carlo method. It then briefly reviews the FDBP decoding algorithm proposed in Ref. [22], which exhibits a higher convergence rate and improved decoding accuracy than conventional binary BP. Finally, it introduces how the combination of FDBP with OSD (FDBP-OSD) is employed to determine the upper bound of code distance based on the Monte Carlo method.

### 3.1. Monte Carlo Method

**Figure 3:**
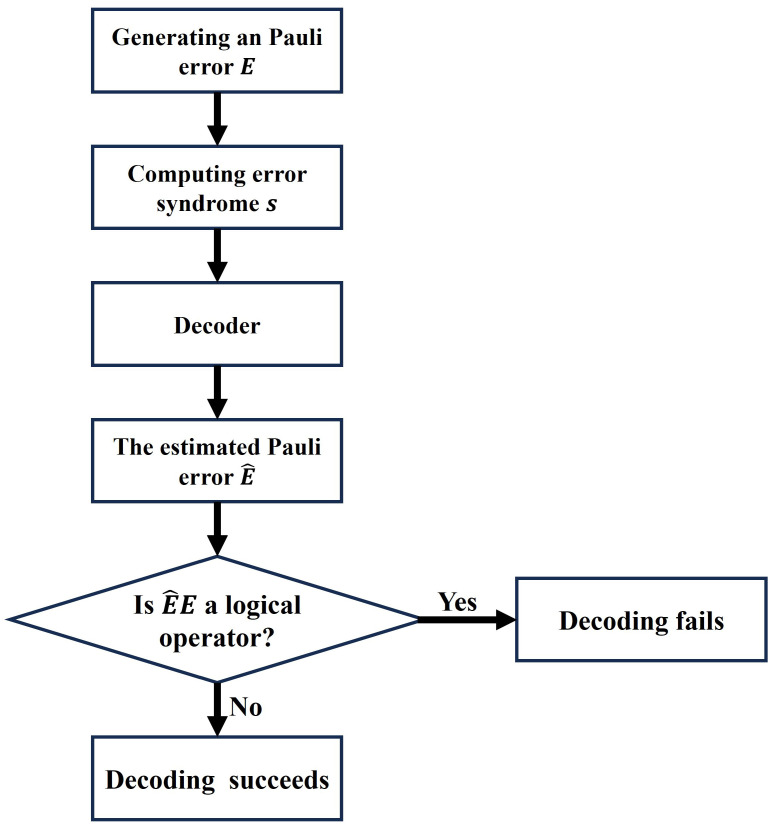
The procedure of quantum error-correction simulations.

To understand the general idea of determining the upper bound of code distance through the Monte Carlo method, it is necessary to comprehend the procedure of quantum error-correction simulations shown in Figure 3. As shown in Figure 3, the first step is to randomly generate a Pauli error *E* and compute the corresponding error syndrome *s*. Then *s* is input into a decoder, and subsequently, the decoder outputs an estimated Pauli error $\hat{E}$ whose corresponding syndrome is also *s*. The last step is to compute $E\hat{E}$; if it is a logical operator, decoding is failed. Otherwise it is a stabilizer, and the decoding procedure succeeds.

An optimal decoder should follow the principle of maximum likelihood decoding. This means it outputs an estimated Pauli error $\hat{E}$ that maximizes the probability of being correct. Under an independent error model, this $\hat{E}$ is likely to have low weight. Furthermore, in the low physical qubit error rate regime, the actual error *E* itself is also likely to be low weight. Consequently, if the combined operator $L=E\hat{E}$ is a non-trivial logical operator (indicating a logical error), it is more probable that *L* has the minimum weight among all equivalent logical operators. This probabilistic tendency forms the basis for determining the upper bound of the code distance using the Monte Carlo method.

This method imposes no strict restrictions on the type of decoder, provided it is applicable to the QSC under test. However, particularly when designing a new QSC, it may not be clear which decoders are applicable. Thus, a decoder offering both high generality and performance is crucial. The combination of FDBP [22] with OSD (FDBP-OSD) provides a highly general and high-performance decoder, making it suitable for determining the upper bound of the code distance for QSCs.

### 3.2. Fully Decoupled Belief Propagation

FDBP, which was proposed in our previous work [22], is an improved BP decoder for QSCs. It achieves higher convergence rates and decoding accuracy than conventional binary BP [23,24].

In conventional binary BP for QSCs, the symplectic representation of Pauli operators causes Pauli-*Y* errors to introduce correlations between vectors $e_{x}$ and $e_{z}$, degrading decoding performance. To address this, prior work proposed a binary BP method leveraging X/Z correlations [28]. However, this approach is limited to CSS codes. For non-CSS codes—especially those under *Y*-biased noise—conventional symplectic binary BP (even with OSD) delivers unsatisfactory results.

To resolve this limitation, we propose FDBP in our previous work [22]. This decoder eliminates *Y*-error-induced correlations between $e_{x}$ and $e_{z}$ in the symplectic representation, making it applicable to both CSS and non-CSS codes. FDBP incorporates three key modifications:

(1) Decoupling Pauli representation: We encode single-qubit Pauli operators using three bits (Definition 1), decoupling *Y*-error correlations.
(2) Decoupled parity-check matrix: Based on (1), we construct a decoupled parity-check matrix (Definition 2)
(3) Constraint message passing: The representation $e=(e_{x}^{\prime }|e_{z}^{\prime }|e_{y}^{\prime })$ enforces $e_{i}+e_{i+n}+e_{i+2n}\leq 1$ for any qubit i≤n. We modify message-update and hard-decision rules to incorporate this constraint.

Simulations in [22] confirm FDBP’s superior convergence rate and accuracy over conventional binary BP. Further implementation details are available therein.

**Definition** **1.** **(Decoupling representation of Pauli operators [** 22 **]).** *The representation which represents Pauli operators by the following mapping is called decoupling representation.*

(14) $$\begin{array}{cc} \; & I\to (0,0,0),\;X\to (1,0,0),\\ \; & Z\to (0,1,0),\;Y\to (0,0,1) \end{array}$$

*For a Pauli error E acting on n qubits, according to the above mapping, its decoupling representation is a binary vector e with size of 3n, namely*

(15) $$e=(e_{x}^{\prime }|e_{z}^{\prime }|e_{y}^{\prime })$$

*where $e_{x}^{\prime }$, $e_{z}^{\prime }$, and $e_{y}^{\prime }$ are all binary vectors with a size of n. Taking $X_{1}Y_{2}Z_{3}$ as an example, the corresponding decoupling representation is $e=\left(e_{x}^{\prime }|e_{z}^{\prime }|e_{y}^{\prime }\right)=\left(1\;0\;0|0\;0\;1|0\;1\;0\right)$.*

**Definition** **2.** **(Decoupled parity-check matrix [**22**]).** *Given an [[n,k]] QLDPC code C and the symplectic representation of its stabilizer generators $H=(H_{x}|H_{z})$, the decoupled parity-check matrix of C is*

(16) $$H_{d}=\left(H_{z}|H_{x}|\left(H_{x}⊕H_{z}\right)\right)$$

*whose dimension is (n−k)×3n and where* ⊕ *denotes addition modulo 2. We can see that given the decoupling representation $e=(e_{x}^{\prime }|e_{z}^{\prime }|e_{y}^{\prime })$ of a Pauli error E and the decoupled parity-check matrix $H_{d}=\left(H_{z}|H_{x}|\left(H_{x}⊕H_{z}\right)\right)$ of a QSC, the error syndrome s is*

(17) $$s=(H_{d}\cdot e)\;mod\;2$$

### 3.3. Algorithm

Algorithm 1 presents the use of FDBP-OSD to determine the upper bound on the code distance. The core idea involves exploiting FDBP-OSD to perform error-correction simulations repeatedly under varying physical qubit error rates, which increases the likelihood that the estimated upper bound converges to the true code distance. The noise model used in most of our simulations is the depolarizing noise model; namely, for a given error rate *p*, one of the Pauli error, *X*, *Y*, and *Z*, acts independently on a qubit with probability $\frac{p}{3}$. This is because we aim to determine the upper bound on the code distance of QECs rather than the upper bound on the effective code distance. Thus, the depolarizing noise model is the most appropriate choice.

**Algorithm 1:** Determining the upper bound of code distance based on FDBP-OSD. 
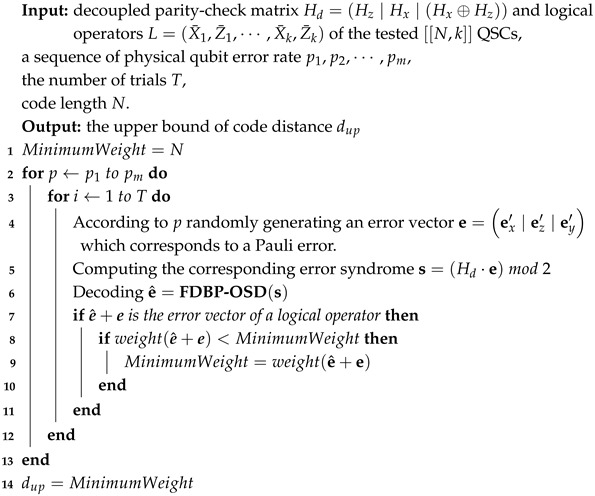


## 4. Simulation Results

In this section, we employ Algorithm 1 to first compute upper bounds on the code distance for three well-characterized codes: the planar surface code [5,6], the XZZX surface code [7], and the toric code [8]. This verifies the algorithm’s effectiveness. Second, we simulate Z-TGRE codes proposed in our prior work [25]. Third, we explore the upper bound for Chamon codes—constructed as the XYZ product of three repetition codes (block lengths $n_{1}$, $n_{2}$ and $n_{3}$)—whose true code distance remains unknown.

**Figure 4:**
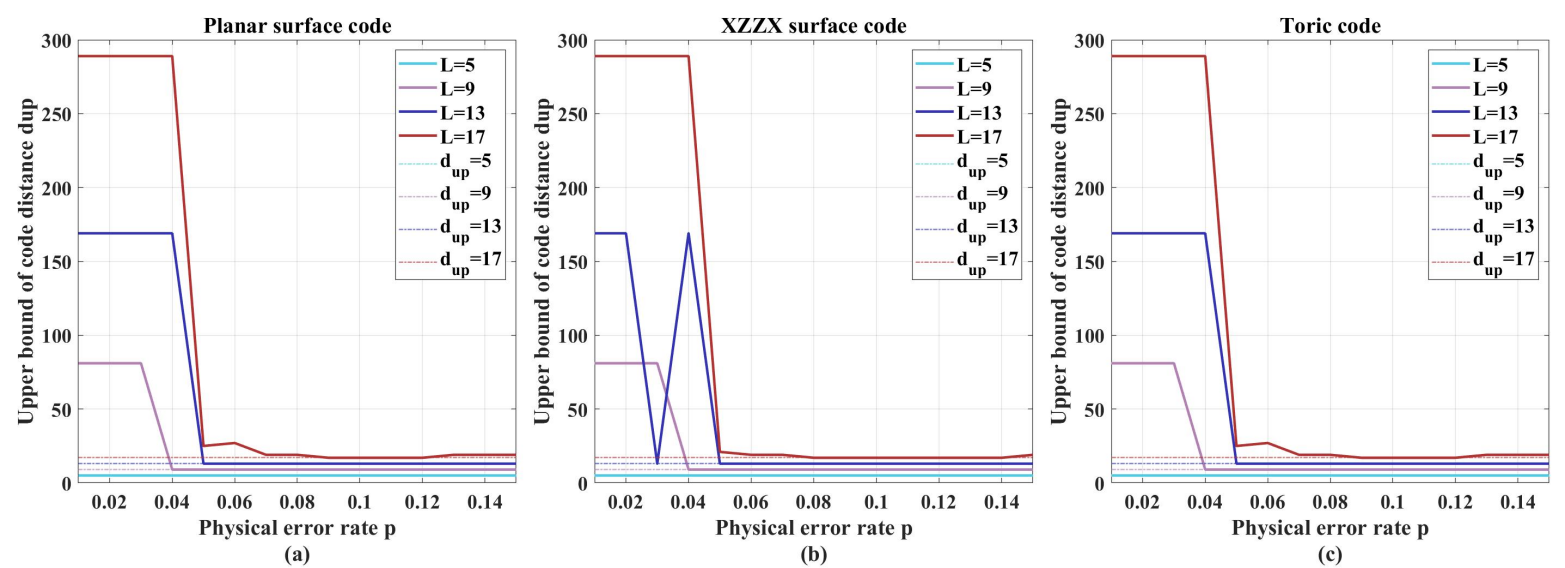
The upper bound on the code distance of (**a**) the planar surface code, (**b**) the XZZX surface code, and (**c**) the toric code, respectively, which are determined by Algorithm 1 using different physical qubit error rates.

Figure 4a–c show the upper bounds determined by Algorithm 1 at varying physical qubit error rates for the planar surface, XZZX surface, and toric codes, respectively. Notably,

(1) The computed upper bounds match the known code distances, demonstrating high algorithm precision.
(2) At low error rates, the upper bound equals the code length (used as the initial value in simulations). This occurs because the high performance of FDBP-OSD ensures all decoding trials succeed.
(3) Simulations indicate that near p=0.1, the algorithm more reliably converges to upper bounds close to the true code distance.

**Figure 5:**
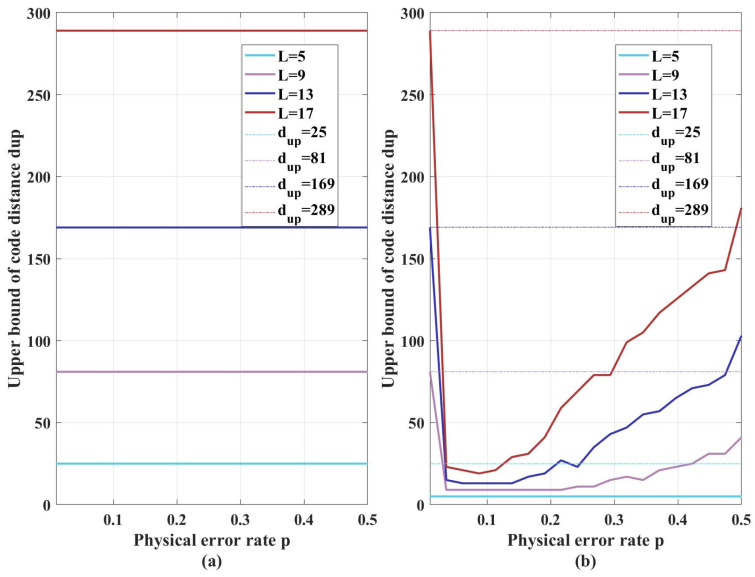
The upper bound on the effective code distance under pure Pauli-Y noise obtained by (**a**) FDBP-OSD and (**b**) BP-OSD.

In addition, to demonstrate that FDBP-OSD possesses better performance under Pauli-*Y*-biased noise compared to conventional BP-OSD, Figure 5a,b show the upper bound of the effective code distance under pure Pauli Y noise obtained by FDBP-OSD and BP-OSD, respectively. One can see that the effective code distance of the XZZX surface code under pure Pauli-*Y* noise obtained by FDBP-OSD equals to the code length, which is fully consistent with the fact that the XZZX surface code under pure Pauli-*Y* noise is equivalent to a repetition code. In contrast, the effective distance obtained by conventional BP-OSD is much smaller than the code length. This is because conventional BP-OSD cannot handle the correlations induced by Pauli *Y* errors, leading to results inconsistent with theoretical expectations. This outcome further demonstrates the advantage of FDBP-OSD in determining the upper bound on the code distance.

In Ref. [25], we propose a new class of QSCs, Z-TGRE codes, with a coding rate of $\frac{1}{2}$, which can only correct pure Pauli-X or -Y errors. Table 1 shows the minimum weight of logical *X* operators determined by Algorithm 1 (right column) of Z-TGRE codes with different code lengths, which is consistent with the theoretical analysis (middle column).

Table 2 shows that, for the Chamon code, which is the XYZ product of three repetition codes with lengths $n_{1}$, $n_{2}$, and $n_{3}$ when $n_{1}=n_{2}=n_{3}=L$, its upper bound of code distance is 2L, and when $n_{1}=L-1$, $n_{2}=L$, and $n_{3}=L+1$, its upper bound of code distance is L(L−1). The results imply that the code distance of XYZ product codes can very likely achieve $O(N^{2/3})$, which supports the conjecture by Leverrier et al. [26].

## 5. Conclusions

The code distance is a critical parameter of QSCs, determining the number of errors that can be reliablly corrected. However, exact computation of the code distance is NP-complete. Consequently, we can compute its upper bound using more efficient methods. This work leverages the Monte Carlo method—an approach for efficient stochastic approximation of complex problems—to propose an algorithm for determining upper bounds on the code distance of QSCs via FDBP-OSD. Our results demonstrate the algorithm’s effectiveness: (1) For the planar surface code, the XZZX surface code, and the toric code, the computed upper bounds match their known code distances, confirming high precision. (2) For the Z-TGRE code, the algorithm-determined upper bound on the minimum weight of logical *X* operators aligns with theoretical analysis. (3) For the Chamon code, the XYZ product of three repetition codes with block lengths $n_{1}$, $n_{2}$, and $n_{3}$ is found. When $n_{1}=n_{2}=n_{3}=L$, its upper bound of code distance is 2L, and when $n_{1}=L-1$, $n_{2}=L$, and $n_{3}=L+1$, its upper bound on code distance is L(L−1). These results suggest that the code distance scales as $O(N^{2/3})$, which supports the conjecture by Leverrier et al. [26]. Crucially, FDBP-OSD’s high generality, performance, and speed for both CSS and non-CSS codes make this algorithm a practical tool for rapidly estimating the upper bound on code distance in novel QSC designs where exact computation is infeasible.

## Figures and Tables

**Table 1:** The minimum weight of logical *X* operators $wt_{min}\left(\bar{X}\right)$ (theoretical and found by Algorithm 1) of Z-TGRE codes with code length *N* from 4 to 512.

Code Length N	${\mathrm{wt}}_{\min}\left(\bar{X}\right)$ Theoretical	${\mathrm{wt}}_{\min}\left(\bar{X}\right)$ Determined by Algorithm 1
4	2	2
8	4	4
16	4	4
32	6	6
64	6	6
128	8	8
256	8	8
512	10	10

**Table 2:** The upper bound of code distance of Chamon codes, which is the XYZ product of 3 repetition codes with lengths $n_{1}$, $n_{2}$, and $n_{3}$.

$n_{1}$	$n_{2}$	$n_{3}$	Code Length *N*	The Upper Bound of Code Distance
2	2	2	32	4
3	3	3	108	6
4	4	4	256	8
5	5	5	500	10
2	3	4	96	6
3	4	5	240	12
4	5	6	480	20

## Data Availability

All the data generated or analyzed throughout this study are included in this article.

## Abbreviations

The following abbreviations are used in this manuscript:

QSC	quantum stabilizer code
Z-TGRE	Z-type Tanner-graph-recursive-expansion
CSS	Calderbank–Shor–Steane
QLDPC	quantum low-density parity check
BP	belief propagation
FDBP	fully decoupled belief propagation
OSD	ordered statistics decoding
